# Advances in Surface-Enhanced Raman Scattering Sensors of Pollutants in Water Treatment

**DOI:** 10.3390/nano13172417

**Published:** 2023-08-25

**Authors:** Grégory Barbillon, Hélène Cheap-Charpentier

**Affiliations:** 1 EPF-Ecole d’Ingénieurs, 55 Avenue du Président Wilson, 94230 Cachan, France; helene.cheap-charpentier@epf.fr; 2 Laboratoire Interfaces et Systèmes Electrochimiques, Sorbonne Université, CNRS, UMR 8235, LISE, 4 Place Jussieu, 75005 Paris, France

**Keywords:** heavy ions, pesticides, pharmaceuticals, sensors, SERS, water treatment

## Abstract

Water scarcity is a world issue, and a solution to address it is the use of treated wastewater. Indeed, in these wastewaters, pollutants such as pharmaceuticals, pesticides, herbicides, and heavy ions can be present at high concentrations. Thus, several analytical techniques were initiated throughout recent years for the detection and quantification of pollutants in different types of water. Among them, the surface-enhanced Raman scattering (SERS) technique was examined due to its high sensitivity and its ability to provide details on the molecular structure. Herein, we summarize the most recent advances (2021–2023) on SERS sensors of pollutants in water treatment. In this context, we present the results obtained with the SERS sensors in terms of detection limits serving as assessment of SERS performances of these sensors for the detection of various pollutants.

## 1. Introduction

Nowadays, the decrease of the supply in water is a world issue due to the pollution of water. Moreover, various factors such as climate changes, population increase, and industrialization increase the demand in water [1,2]. Thus, alternate sources of water are currently in investigation to sustainably manage water [3,4]. In addition, the appearance of wastewater treatment can allow obtaining a supplementary supply source in water, which can reduce the water scarcity [5,6]. Nevertheless, wastewater reuse is highly dependent on suitable treatments following the requirements of the water quality. In these requirements, a major worry concerns the complete or partial removal of compounds of interest in order to preserve the environment and keep public health safe [7,8]. The compounds of interest mainly detected at strong concentrations in different types of water (wastewater, surface water, groundwater, seawater, and tap water) are pesticides, insecticides, personal care products, pharmaceuticals, and heavy metals [9]. These compounds in water come in major part from discharges of wastewater treatment plants where they are badly eradicated [10,11]. To boost the elimination performances of these compounds before discharge, several treatments of a biological, chemical, or physical nature have been investigated [12,13]. In parallel, it is important to obtain the detection and quantification of all these compounds in different types of water and also during the treatment of the latter by using quick, precise, and robust analytical techniques [14]. The most widely used techniques for detection and quantification are capillary electrophoresis, gas chromatography mass spectrometry, and high-performance liquid chromatography mass spectrometry [15,16,17,18]. Nonetheless, these techniques have a high cost and they are time-consuming in terms of sample preparation [18,19]. Additionally, colorimetric, electrochemical, and fluorescence sensors are also employed for the detection of these compounds of interest for water treatment and more widely for the environment [20,21,22,23,24]. These sensors are easy to use, portable, and accurate [22]. However, the performances of these need to be demonstrated in a complex environment. Furthermore, surface-enhanced Raman scattering (SERS) sensors are also used for the detection of these compounds [25,26,27,28,29]. This technique is very sensitive and gives details on the molecular structure. Thus, it can specifically recognize particular biomolecules as the compounds of interest cited previously.

In this mini-review, the goal is reporting on the most recent advances (2021–2023) in SERS sensors for the detection of compounds of interest (pollutants) in water treatment. Moreover, various review articles on the detection of pollutants for environmental analyses (e.g., water treatment) are already available in the scientific literature, but with different focuses [30,31,32,33,34]. Herein, we will centralize the SERS sensors of pesticides, herbicides, and organic dyes for water treatment in the first part; the second one concerns the detection of pharmaceuticals, personal care products, and heavy ions.

## 2. SERS Sensors of Pollutants in Water Treatment

SERS sensing is not an issue in water because the targeted molecules (here pollutants) may be detected within the water. Thus, the SERS technique is a perfect tool allowing a quick sensing of various molecules in water [35,36]. The SERS effect is based on the enhancement of the Raman signal (vibration modes) of molecules thanks to plasmonic nanosystems [37,38,39,40]. The enhancement can mainly be due to two contributions coming from electromagnetic and chemical interactions between molecules and plasmonic nanosystems [41,42,43]. For the electromagnetic contribution, the enhancement is due to highly confined electric fields, named hotspots in the literature [44,45,46,47], when the plasmon excitations in metallic nanosystems match the excitation wavelength used for Raman experiments [42,43,48,49,50]. For the chemical contribution, the enhancement is mainly due to charge-transfers between the plasmonic/hybrid nanosystems and molecules [41,43,51,52,53]. Here, we use the limit of detection (LOD) for the assessment of SERS performances of sensors.

### 2.1. Detection of Pesticides, Herbicides, and Organic Dyes

Here, the detection of pollutants such as pesticides, herbicides, and organic dyes for water treatment is addressed (see Table 1). We start with the detection of organic dyes [54,55,56,57,58,59,60], then those of pesticides [61,62,63,64,65,66], and finally those of herbicides [67,68].

Concerning to the detection of organic dyes, Li et al. have reported a LOD of $8.7\times 10^{-10}$ M for MG detection in fishpond water [54]. For this experiment, a SERS substrate based on a densely packed monolayer of plasmonic Au@Ag nanocuboids was carried out by this research group. In this monolayer, the edges and corners of nanocuboids allowed huge SERS performances by generating many hotspots [54]. Furthermore, the group of Song developed another strategy of SERS substrate by employing nanostructures of aluminum oxide hydroxide decorated with silver nanoparticles (AlOOH@Ag) for the detection of the Congo Red (CR) molecules in river water and industrial wastewater [55]. LODs of $10^{-9}$ M for CR molecules have been recorded in these two types of water with AlOOH@Ag nanostructures, and owing to their porosity, the density of molecules (here: CR) present in the area of hotspots of silver nanoparticles was, thus, highly improved [55]. Another approach was used by Yang et al., where they demonstrated excellent LODs of $10^{-12}$ M and $10^{-11}$ M for MG detection in water coming from the Fuxian and Dian lakes, respectively [56]. The authors fabricated and used a SERS substrate consisting of a TiO${}_{2}$ flower-like nanomaterial decorated with silver nanoparticles. They reported that the enhancement of the Raman signal was due to the generation of a larger number of hotspots from the obtained SERS substrate and also the charge transfer mechanism of the molecule/semiconductor/metal system [56]. In another way, Kang et al. showed an interesting SERS substrate, and this consisted of strongly porous gold supraparticles. The authors detected malachite green isothiocyanate (MGTIC) molecules in wastewater with a LOD of $10^{-8}$ M by using these supraparticles, and the SERS enhancement came from the presence of interstitial gaps between gold nanoparticles (hotspots) [57].

**Figure 1:**
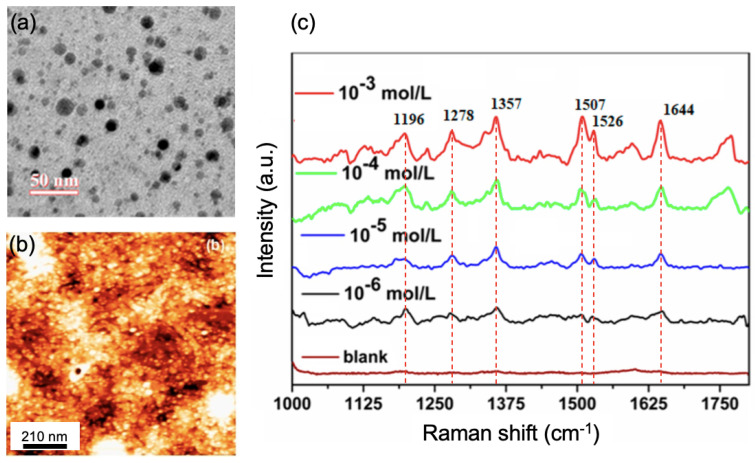
(**a**) TEM picture of the AgNP-PS-*b*-PAA film. (**b**) AFM picture of the AgNP-PS-*b*-PAA film. (**c**) SERS spectra of the rhodamine B molecules in water, recorded on the AgNP-PS-*b*-PAA film. The Raman spectrum called blank is recorded on PS-*b*-PAA film (without AgNPs) with a rhodamine B concentration of 1 mM. All the figures are reprinted (adapted) with permission from [60], Copyright 2021 American Chemical Society.

Moreover, Liu et al. studied the use of plasmonic sensors composed of silver nanocubes (AgNCs) having an elevated degree of purity for the detection of MG molecules in aquaculture water. Liu et al. assessed the LOD of MG molecules at $2.6\times 10^{-7}$ M in this aquaculture water [58]. This LOD was obtained thanks to the SERS enhancement coming from the coupling between Ag nanocubes (hotspots) and the matching between the excitation wavelength of 785 nm employed for Raman measurements and the plasmon resonance peak corresponding to the monolayer of silver nanocubes [58]. Chen et al. proposed another SERS substrate for the detection of the p-nitroaniline (PNA) in river water. The SERS substrate was achieved by copolymerizing the molecular imprinted polymer (MIP) on a defect-graphene layer with Ag nanoparticles, and they realized a LOD of $2.5\times 10^{-15}$ M for detecting PNA in river water, owing to the presence of hotspots produced by the gaps between AgNPs [59]. To finish this part on the detection of organic dyes, Daripa et al. carried out a SERS substrate composed of a block copolymer film (block copolymer polystyrene-*block*-poly(acrylic acid), called PS-*b*-PAA), which is decorated with silver nanoparticles [60] (see Figure 1a,b). A LOD of $10^{-6}$ M for Rhodamine B detected in water was found (see Figure 1c), which is similar or higher than what has been demonstrated in the literature with this strategy based on a block copolymer. The enhancement of the Raman signal was due to the presence of hotspots generated by the coupling between Ag nanoparticles [60].

**Figure 2:**
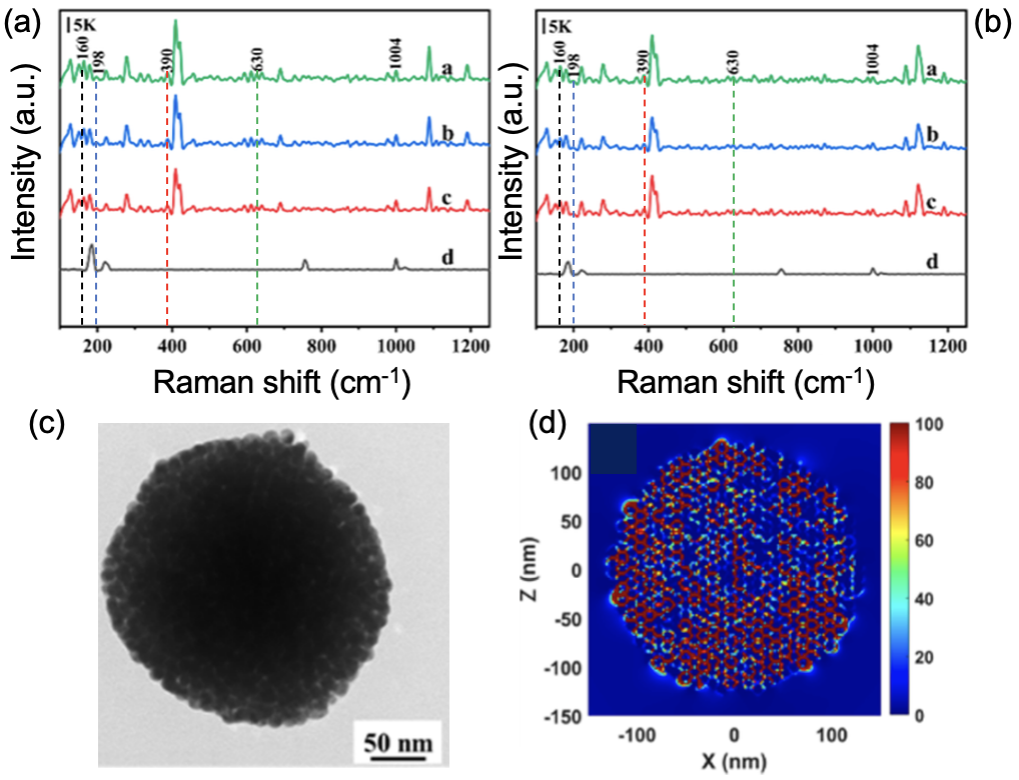
SERS spectra of (**a**) the river water and (**b**) the fishpond water, containing the following OCP: tetradifon (blue dashed line: peak at 198 cm${}^{-1}$), 4,4’-DDT (red dashed line: peak at 390 cm${}^{-1}$), chlordane (green dashed line: peak at 630 cm${}^{-1}$), and α-endosulfan (black dashed line: peak at 160 cm${}^{-1}$). For (**a**,**b**): a,b,c correspond to following OCP concentrations: tetradifon (150 nM, 50 nM, and 5 nM, respectively), 4,4’-DDT (150 nM, 50 nM, and 5 nM, respectively), chlordane (150 nM, 50 nM, and 5 nM, respectively), and α-endosulfan (50 nM, 30 nM, and 6 nM, respectively), and d corresponds to water without OCP. (**c**) TEM picture of a gold superparticle. (**d**) Electric field mapping of a gold superparticle at a wavelength of 785 nm corresponding to the excitation wavelength used for Raman measurements. All the figures are reprinted (adapted) with permission from [61], Copyright 2021 American Chemical Society.

Regarding the detection of pesticides, Shi et al. developed another interesting idea of a SERS substrate for the detection of the pesticide (4-Aminothiophenol: 4-ATP) in river water [62]. This SERS substrate consisted of a TiO${}_{2}$-coated Cu sheet on which metal-organic frameworks (ZIF-67) were deposited, and then Ag nanoparticles. The Ag/ZIF-67/TiO${}_{2}$/Cu sheet allowed reaching a LOD of $5\times 10^{-11}$ M for the detection of 4-ATP molecules in river water. These SERS performances of detection have been obtained thanks to the coupling between Ag nanoparticles inducing hotspots, and also to electron transfers between AgNPs and 4-ATP molecules [62]. Another group has also developed a relatively similar strategy. Indeed, Chen et al. used the following sheet: Ag/ZIF-8/TiO${}_{2}$/Al for the detection of the same pesticide (4-ATP) in river water. A LOD of $10^{-9}$ M for the detection of 4-ATP molecules in river water was found [63]. The SERS performances come from the same phenomena mentioned in the previous example. Moreover, Yao et al. showed a LOD of 1.2 μg/L for the detection of the pesticide paraquat in tap and drinking water. To do that, Yao et al. had a simple approach based on the use of a natural lotus leaf on which Ag nanoparticles were dropped. These Ag nanoparticles were arranged in closely packed arrays on this lotus leaf, thus providing a great number of hotspots, allowing the SERS enhancement and, consequently, the nice LOD obtained [64]. Zhu et al. investigated a film composed of Ag nanocubes/graphene-oxide/Au-nanoparticles (AgNCs/GO/AuNPs) for the SERS detection of the pesticide thiram in drinking water. A LOD of 0.37 μg/L was found with this film due to the SERS enhancement coming from plasmonic hotspots produced between AuNPs and AgNCs/GO [65]. Han et al. developed an on-site SERS detection of the pesticide coumatetralyl (CMTT) in environmental water by using the aggregation of gold nanoparticles via the salt addition of magnesium nitrate (Mg(NO${}_{3}$)${}_{2}$) and with a portable Raman spectrometer [66]. Thus, a LOD of 1.53 μg/L for the detection of CMTT was found thanks to the SERS enhancement resulting from the production of hotspots upon the aggregation of Au nanoparticles [66]. To conclude this part on the detection of pesticides, Li et al. reported on the detection of organochlorine pesticides (OCP) such as tetradifon, dichlorodiphenyltrichloroethane (4,4’-DDT), chlordane, and α-endosulfan in river and fishpond waters [61] (see Figure 2a,b). To do that, Li et al. fabricated colloidal gold superparticles consisting of an assembly of gold nanoparticles of 10 nm (see Figure 2c), which have enabled reaching LODs of 5 nM–6 nM for the four OCPs named previously. The SERS enhancement was achieved thanks to various nanogaps within gold superparticles, thus creating intense hotspots (see Figure 2d) [61].

**Figure 3:**
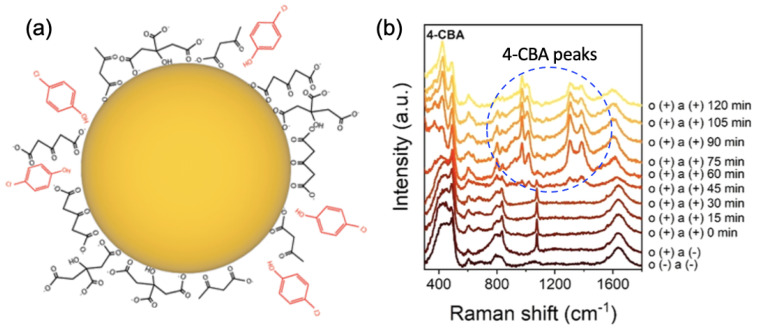
(**a**) Scheme of the Au nanoparticles with the molecules of 4-CBA (in red color). (**b**) SERS spectra of an AuNPs solution with 10 mM of PDS and 57 μM of 4-CBA for several durations under sunlight illumination (oxydation time of the citrate layer). For the caption, “o(+)”, “o(−)”, “a(+)”, and “a(−)” correspond to the presence (+) and absence (−) of PDS (o) and 4-CBA (a), respectively. All the figures are reprinted (adapted) with permission from [68], Copyright 2022 American Chemical Society.

Regarding the detection of herbicides, Yang et al. studied the detection of the herbicide 2,4-dichlorophenoxyacetic acid (2,4-D) in mineral and river waters with a promising SERS substrate [67]. This SERS substrate consisted of silver nanoclusters coated with gum arabic (Ag-GA). These Ag-GA nanoclusters allowed the generation of hotspots coming from the production of nanogaps between these, thus inducing an enhancement of the SERS effect. Moreover, it is well-known that gum arabic can capture an analyte (here 2,4-D) very close to the surface of nanostructures coated with GA. Thus, a LOD of $1.5\times 10^{-10}$ M for the detection of 2,4-D molecules in mineral and river waters was achieved with this SERS substrate [67]. To finish this part concerning to the detection of herbicides, and also this section, Wang et al. developed the following strategy for the detection of the herbicide 4-chlorobenzoic acid (4-CBA) [68] (see Figure 3). They used the oxidation control of the citrate layer for gold nanoparticles in order to improve the SERS effect. This control was realized with sulfate radicals coming from photolysis of peroxydisulfate (PDS), thus permitting the production of a great number of hotspots (aggregation of AuNPs) for the detection of 4-CBA. Thereby, a LOD of $5.7\times 10^{-5}$ M for the detection of 4-CBA in water was found with this strategy (see Figure 3b) [68].

### 2.2. Detection of Pharmaceuticals, Personal Care Products, and Heavy Ions

Herein, the detection of pharmaceuticals, personal care products (PCP), and heavy ions in water treatment is introduced (see Table 2). We begin with the detection of pharmaceuticals and PCP [69,70,71,72,73,74,75,76,77,78,79], and we end with those of heavy ions [80,81,82,83,84].

**Figure 4:**
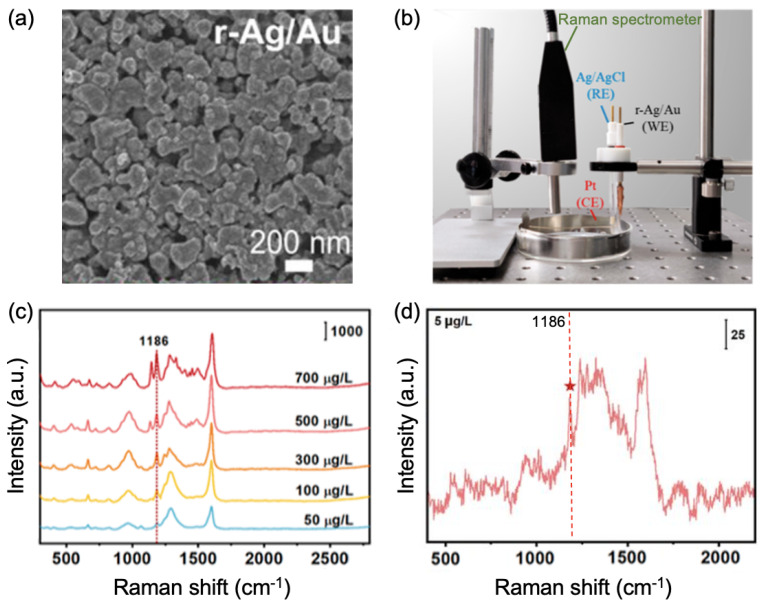
(**a**) SEM picture of the r-Ag/Au fiber morphology. (**b**) Setup photo of the technique combining the three functionalities (working electrode, SPME, and SERS). The WE, RE, and CE electrodes correspond to the working, reference, and counter electrode, respectively. SERS spectra of benzidine recorded on the r-Ag/Au fiber at (**c**) several concentrations, and (**d**) at LOD of 5 μg/L. On (**c**,**d**), the red dashed line and the one with the red five-pointed star correspond to the vibration mode of benzidine located at 1186 cm${}^{-1}$. All the figures are reprinted (adapted) with permission from [70], Copyright 2022 American Chemical Society.

Regarding the detection of pharmaceuticals and personal care products, Haung et al. investigated the sensing of the antibiotic quinoline in wastewater. The authors employed self-assembled gold nanoparticles with highly uniform gaps of 0.9 nm between Au nanoparticles producing various hotspots, thus inducing a good enhancement of the SERS effect. Therefore, a LOD of 5 μg/L for quinoline molecules was reached in wastewater [69]. An alternative technique of the in situ detection of pharmaceuticals and personal care products was developed by Li and co-workers [70]. In this work, a rough Ag fiber coated with an Au layer (r-Ag/Au fiber; see Figure 4a) was employed for this detection. This fiber has three functionalities: (i) solid phase microextraction (SPME), (ii) working electrode, and (iii) SERS substrate. The SPME method allows the extraction of pharmaceuticals and personal care products from the sample to the SPME sorbent (functionalized layer of extraction), and, at the same time, these molecules are detected and analyzed by SERS measurements with a portable Raman spectrometer (see Figure 4b). In addition, the functionality as a working electrode allows an enhanced adsorption for a quick detection. Thus, Li et al. applied this technique with these three functionalities to the detection of benzidine in domestic water (see Figure 4c,d) and they obtained a LOD of 5 μg/L (see Figure 4d). This LOD was reached thanks to the enhancement of the SERS effect coming from the rough surface and deep pores of the r-Ag/Au fiber (see Figure 4a) producing a great number of hotspots [70].

**Figure 5:**
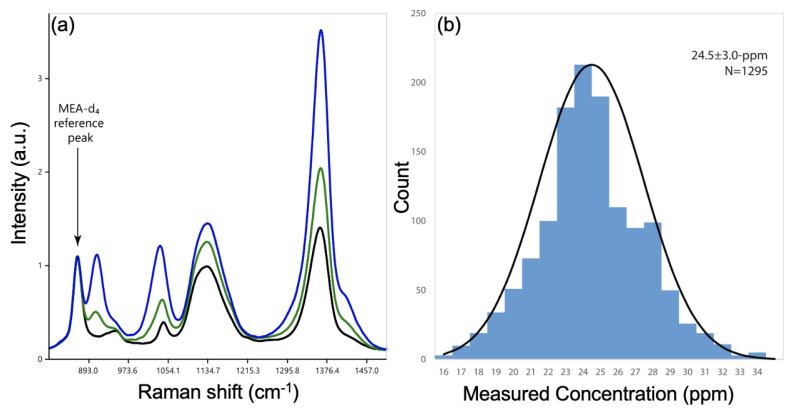
(**a**) SERS spectra of MEA and its isotopologue MEA-d${}_{4}$: in black, 50 ppm of MEA-d${}_{4}$ without MEA, in green with 25 ppm of MEA, and in blue with 75 ppm of MEA. The MEA-d${}_{4}$ peak at 870 cm${}^{-1}$ corresponds to the reference peak. (**b**) Measurements of MEA concentration during a period of 4.5 years collected with 25 distinct Raman spectrometers for an initial concentration of MEA of 25 ppm. All the figures are reprinted (adapted) with permission from [73], Copyright 2023 American Chemical Society.

Another strategy was developed by Berganza et al. for the detection of another antibiotic (ciprofloxacin = CIP) in water. This strategy consisted of the use of magnetite (Fe${}_{3}$O${}_{4}$) nanorods coated with Au nanospheres by concentrating them with the magnetic field of a magnet in a specific location for SERS measurements. By concentring these Fe${}_{3}$O${}_{4}$NR@AuNPs, efficient hotspots were, thus, generated, permitting a sensitive detection of CIP in water. A LOD of $10^{-7}$ M for CIP was reached thanks to these magneto/plasmonic nanorods [71]. In another way, Jaitpal et al. fabricated SERS substrates with a 3D-printing technique for the detection of the genotoxic 5-chloro-2-methyl-4-isothiazolin-3-one (CMIT) in lake water [72]. These SERS substrates consisted of the realization of a copper–polylactic acid disk with 3D printing on which silver microstructures were deposited. As seen previously in several works, effective hotspots were created in the silver microstructures in order to have a good enhancement of the SERS effect, thus allowing a sensitive LOD to be attained. This was assessed at 10 mg/L for CMIT molecules in lake water [72]. By another strategy, Benhabib et al. showed the SERS detection of the monoethanolamine (MEA) in the water of the refinery process [73]. In this study, the authors employed gold nanoparticles for enhancing the SERS signal, but also an internal standard, which is an isotopologue of MEA, which is structurally identical, but can be distinguished in a spectroscopic manner (see Figure 5a). The use of this isotopologue in a given quantity allowed a distinct Raman signal serving as reference to be obtained, thus permitting a comparison of all the samples (see Figure 5a). This method does not require calibration and is stable for several years (see Figure 5b). With this method, the authors reached a LOD of 1.8 mg/L for MEA in the water of the refinery process [73].

Burtsev et al. showed an SERS detection of ibuprofen in water by using gold multibranched nanoparticles (AuMNPs) coupled to an extraction process, both in a microfluidic chip [74]. The principle of detection developed by Burtsev et al. is based on the extraction of ibuprofen from water to the organic phase by employing a mixer in the microfluidic chip, followed by the addition of lipophilically functionalized AuMNPs in this organic phase. Thus, these AuMNPs caught ibuprofen after its extraction and detection by SERS measurements. Moreover, these AuMNPs have the advantage of producing effective hotspots, inducing an efficient enhancement of the SERS effect. Therefore, a LOD of $10^{-8}$ M for ibuprofen was achieved in water [74]. Another strategy developed by Zhang et al. was employed for the SERS detection of the opioid fentanyl in wastewater [75]. This consisted of the fabrication of silver nanoparticles on a diatomaceous earth (DE) film. The use of this DE film has allowed supplementary hotspots produced by its two-dimensional pores. Therefore, the enhancement of the SERS signal was due to hotspots of the DE film and silver nanoparticles. As a result, a LOD of 0.8 μg/L was achieved for fentanyl molecules in wastewater [75]. Moreover, Lê et al. investigated the SERS detection of polystyrene microplastic particles (PMPP) in tap, river, and sea water [76]. To do that, the authors fabricated gold nanostars coated with silver included in the nanopores of anodized aluminum oxide (AuNSs@Ag@AAO). The AuNSs@Ag produced hotspots located at nanoparticle tips, but also in nanogaps realized between the AuNSs@Ag in AAO nanopores, thus allowing a high efficiency of SERS signal enhancement. The recorded LOD with these AuNSs@Ag@AAO templates was 50 mg/L of PMPP, the size of which was 400 nm [76]. In another way, Mao et al. demonstrated a sensitive SERS detection of methamphetamine (MA) in wastewater [77]. Mao et al. realized core(Au)-shell(Ag) nanoparticles (Au@AgNPs) on a glass nanofibrous paper (GNFP). The three-dimensional high porosity of GNFP allowed an increase in the surface on which Au@AgNPs were deposited. Therefore, a great number of hotspots was generated, inducing a huge enhancement of the SERS signal. As a result, a LOD of 7.2 ng/L for MA molecules was found in wastewater [77]. Therewith, Zhang et al. demonstrated the on-site SERS detection of coronavirus (SARS-CoV-2) in 23 types of water by using an array of silver nanorods functionalized with the angiotensin converting enzyme 2 (ACE2) [78]. The interest of employing the functionalization with ACE2 is to catch SARS-CoV-2 from water samples. The detection of SARS-CoV-2 was observed when the SERS intensity for a major part of Raman peaks of ACE2 was decreased upon the catching of SARS-CoV-2 spike proteins on the ACE2@AgNRs array, indicating a positive test to coronavirus. The enhancement of the SERS signal was due to the hotspots coming from plasmonic modes of the ACE2@AgNRs array upon the excitation with a laser used for Raman measurements [78]. To finish this part on the detection of of pharmaceuticals and personal care products, Palermo et al. showed an SERS detection of the virus of hepatitis A (HAV) in water by using a metasurface composed of pyramidal nanoholes in gold film. For this plasmonic metasurface, the enhancement of the SERS signal was due to the presence of hotspots within cavities and in the gaps between cavities. As a result, a LOD of 13 ng/L was achieved for the detection of HAV in water [79].

**Figure 6:**
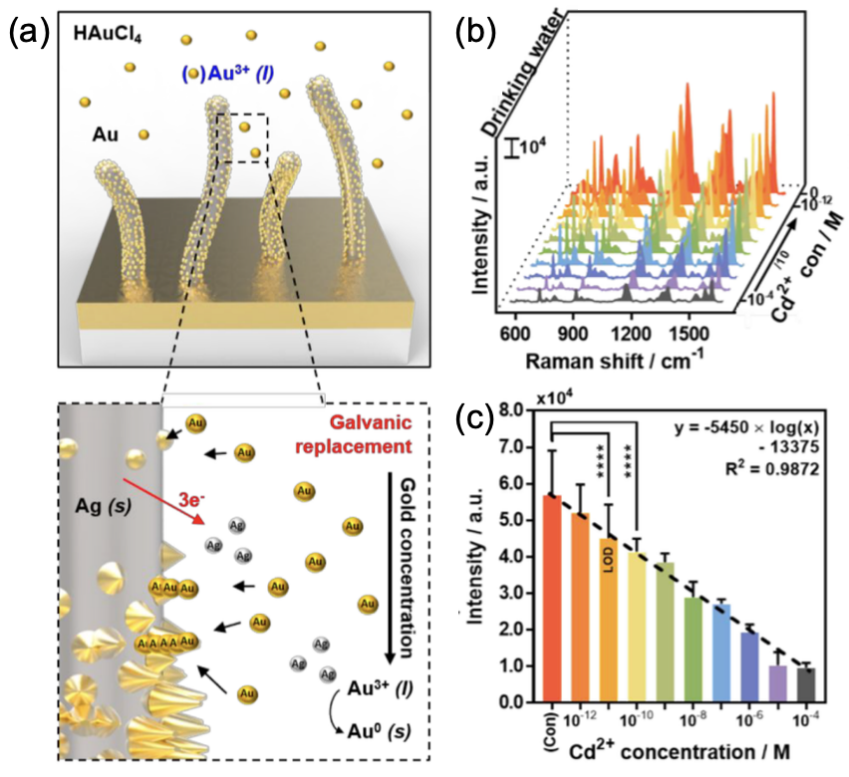
(**a**) Illustration of the NPP-NS fabrication. (**b**) SERS spectra obtained with NPP-NS in drinking water mixed with a CV solution containing Cd${}^{2+}$ ions at concentrations ranging from $10^{-12}$ M to $10^{-4}$ M, and a control without Cd${}^{2+}$ ions (only with CV). (**c**) SERS intensity at 1175 cm${}^{-1}$ versus Cd${}^{2+}$ concentration (M), where (Con) corresponds to the control solution of CV without Cd${}^{2+}$ ions. All the figures are reprinted (adapted) with permission from [83], Copyright 2022 American Chemical Society.

Concerning the detection of heavy ions, Zorlu et al. investigated the SERS detection of the heavy ion Cu${}^{2+}$ in tap water [80]. To do that, the authors employed polystyrene beads on which Ag nanoparticles were deposited; then, metal–organic frameworks (ZIF-8) were added. The principal advantage to use ZIF-8 was to improve the SERS signal due to a better matching of the plasmonic resonance of these nanoparticles (PS@Ag@ZIF-8) that was redshifted (increase of the local refractive index) and the excitation wavelength used for Raman spectroscopy. The PS@Ag@ZIF-8 nanoparticles generated hotspots in the nanogaps between AgNPs. Moreover, the affinity of ZIF-8 for a selective molecule for copper (bathocuproine) allowed an efficient and selective detection of copper ions (Cu${}^{2+}$). As a result, a LOD of $10^{-7}$ M for Cu${}^{2+}$ in tap water was observed [80]. With an alternative strategy, He et al. developed a method using a double enhancement for the SERS detection of the heavy ion Pb${}^{2+}$ in water [81]. He et al. fabricated an SERS substrate consisting of a porous gallium nitride (GaN) substrate on which Ag nanoparticles were deposited, then Au nanoparticles, and finally a graphene monolayer. This SERS substrate allowed a chemical enhancement (charge transfers between molecules and graphene) and an electromagnetic enhancement (hotspots generated in Au-Ag nanostructures). This Gr/Au/Ag/GaN substrate was functionalized with the molecule of cyanine (Cy3), permitting the quantitative detection of Pb${}^{2+}$ ions. As a result, the LOD was assessed at $4.3\times 10^{-12}$ M for the detection of Pb${}^{2+}$ ions in water [81]. Another idea of SERS substrates has emerged for the detection of Hg${}^{2+}$ ions in lake water, realized by Xu and coworkers [82]. This SERS substrate was based on silver nanoparticles, which were functionalized with molecules of phenylacetylene (Ph). The detection principle is based on the strong decrease of the SERS intensity of the Raman peak located at 1988 cm${}^{-1}$, corresponding to the stretch of the binding (C≡≡≡≡≡≡≡≡C) when Ph molecules were adsorbed on the surface of Ag nanoparticles. Indeed, in the presence of Hg${}^{2+}$ ions, the alkynyl of Ph molecules coordinated to Hg${}^{2+}$ ions by separating from the surface of Ag nanoparticles and, consequently, from hotspots. This induced a decrease of the SERS intensity of the peak at 1988 cm${}^{-1}$. By using this principle, a LOD of $8.8\times 10^{-11}$ M for Hg${}^{2+}$ ions in lake water was reached [82]. In addition, Kim et al. studied the detection of the heavy ion Cd${}^{2+}$ in drinking water by employing biomimetic nanostructures of type nano-pine-pollen (NPP-NS; see Figure 6a) [83]. These NPP-NS were composed of primary Ag nanostructures on which secondary Au nanoparticles were deposited (see Figure 6a). These Au nanoparticles allowed the production of hotspots coming from nanogaps formed by the closely packed organization of AuNPs on Ag nanostructures. Thereby, a LOD of $10^{-11}$ M for Cd${}^{2+}$ in drinking water was reached (see Figure 6b,c). To determine this LOD, the authors recorded the variations of the SERS intensity of the Raman peak at 1175 cm${}^{-1}$, which is characteristic of the molecule of crystal violet (CV) that reacts specifically with Cd${}^{2+}$ ions, by inducing a decrease of the SERS intensity as soon as the CV–Cd${}^{2+}$ complex appeared [83].

To conclude this part on the detection of heavy ions and, more generally, this mini-review, Logan et al. developed a technique of SERS detection of Hg${}^{2+}$ ions in seawater thanks to catalytic gold nanostars functionalized with polyethylene glycol (AuNSs-PEG) [84]. The principle of detection relies on the fact that these AuNSs-PEG catalyzed the oxidation of the molecule of 3,3${}^{\prime }$,5,5${}^{\prime }$-tetramethybenzidine (TMB), giving a product that has huge Raman activity (oxTMB) measured by these same AuNSs-PEG used as the SERS substrate. When Hg${}^{2+}$ ions were added, the SERS intensity of oxTMB was strongly decreased. This decrease was due to the strong alteration of sharpened tips of AuNSs-PEG caused by the appearance of the Au-Hg amalgam, thus significantly reducing the hotspots generated by the initial sharpened tips. By employing this principle, the authors determined a LOD of 0.2 μg/L for Hg${}^{2+}$ ions in seawater [84].

## 3. Conclusions and Outlook

To summarize, in this mini-review, we have described the most recent advances of SERS sensors of pollutants in water treatment. Due to the performances of these SERS sensors, limits of detection were achieved ranging from $2.5\times 10^{-15}$ M to $5.7\times 10^{-5}$ M for the detection of pesticides, herbicides, and organic dyes, and from $4.3\times 10^{-12}$ M to $10^{-7}$ M for the detection of pharmaceuticals, personal care products, and heavy ions. These limits of detection were obtained thanks to the enhancement of the SERS signal coming from the hotspots generated by the different plasmonic nanostructures depicted in the main text or from charge transfer processes or from both. Nonetheless, there remain important areas to be improved upon concerning the SERS sensors of pollutants in different types of water. Indeed, the reproducibility, cost of fabrication, interference minimization from environmental matrices, and reusability and multifunctionality of SERS substrates are the principal key points in order to upgrade the SERS performances of detection. A possibility for minimizing the interference is to use advanced strategies of sample purification. Regarding the reusability and multifunctionality, flexible SERS substrates can be used such as paper- and cellulose-based SERS substrates, permitting the detection on complex surfaces, and other substrate types can be employed, such as SERS substrates with the functionality of self-calibration allowing a detection in complex matrices without any pre-treatment, SERS substrates with the functionality of regeneration giving the possibility of using them several times, and SERS substrates with the functionality of separation allowing a detection in complex matrices by separating the molecule of interest from matrices. Regarding the reproducibility and the cost of the fabrication of SERS substrates, fabrication techniques at a large scale and low cost are now used, but novel materials at a low cost and high reproducibility enhancing the SERS effect at identical magnitudes to noble metals are still to be developed, such as organic/inorganic semiconductors [85] and organic semiconductors with or without a plasmonic layer [86]. Lastly, an interesting step is to develop SERS substrates composed of several functionalities depicted previously, and a couple of groups have already started the investigation of such materials [87,88,89,90].

## Figures and Tables

**Table 1:** SERS substrates, sample, pollutants, and limit of detection (LOD) for SERS sensors (Au@Ag NCs = Gold/silver nanocuboids; MG = Malachite green; AlOOH@Ag = Aluminum oxide hydroxide with silver nanoparticles; FLNM = flower-like nanomaterials; PAuSPs = Porous gold supraparticles; MGTIC = Malachite green isothiocyanate; AgNCs = Silver nanocubes; DG/Ag-MIPs = Defect-graphene/Ag nanoparticles/molecular imprinted polymer; PNA = p-nitroaniline; AgNP-PS-*b*-PAA = Amphiphilic block copolymer polystyrene-*block*-poly(acrylic acid) decorated with Ag nanoparticles; AuSPs = Gold superparticles; OCP = Organochlorine pesticides; ZIF-67 or ZIF-8 = Zeolitic imidazolate framework (-67 or -8); 4-ATP = 4-Aminothiophenol; AgNPs = Silver nanoparticles; AgNCs/GO/AuNPs = Silver nanocubes/graphene-oxide/gold nanoparticles; AuNPs = Gold nanoparticles; CMTT = Coumatetralyl; Ag-GA = Silver-gum arabic; 2,4-D = 2,4-dichlorophenoxyacetic acid; 4-CBA = 4-chlorobenzoic acid).

SERS Substrates	Sample	Pollutants	LOD	Refs.
Au@Ag NCs	Fishpond water	MG	$8.7\times 10^{-10}$ M	[54]
AlOOH@Ag	River, industrial wastewater	Congo Red	$10^{-9}$ M	[55]
TiO${}_{2}$/Ag FLNM	Lake waters	MG	$10^{-12}$ M	[56]
PAuSPs	Wastewater influent	MGITC	$10^{-8}$ M	[57]
AgNCs	Aquaculture water	MG	$2.6\times 10^{-7}$ M	[58]
DG/Ag-MIP	River water	PNA	$2.5\times 10^{-15}$ M	[59]
AgNP-PS-*b*-PAA	Water	Rhodamine B	$10^{-6}$ M	[60]
AuSPs	River, fishpond water	OCP	$5\times 10^{-9}$ M	[61]
Ag/ZIF-67/TiO${}_{2}$/Cu	River water	4-ATP	$5\times 10^{-11}$ M	[62]
Al-TiO${}_{2}$-ZIF-8-Ag	River water	4-ATP	$10^{-9}$ M	[63]
AgNPs	Tap and drinking water	Paraquat	1.2 μg/L	[64]
AgNCs/GO/AuNPs	Drinking water	Thiram	0.37 μg/L	[65]
AuNPs	Environmental water	CMTT	1.53 μg/L	[66]
Ag-GA	Mineral or river water	2,4-D	$1.5\times 10^{-10}$ M	[67]
AuNPs	Water	4-CBA	$5.7\times 10^{-5}$ M	[68]

**Table 2:** SERS substrates, sample, pollutants, and limit of detection (LOD) for SERS sensors (AuNPs = Gold nanoparticles; r-Ag/Au fiber = Rough Ag fiber with Au layer; Fe${}_{3}$O${}_{4}$NR = Magnetite nanorods; CIP = Ciprofloxacin; Ag–Cu–PLA disks = silver microstructures–copper–polylactic acid disks; CMIT = 5-chloro-2-methyl-4-isothiazolin-3-one; MEA = Monoethanolamine; AuMNPs = gold multibranched nanoparticles; AgNPs = Silver nanoparticles; DE film = Diatomaceous earth film; AuNSs@Ag@AAO = gold nanostars coated with silver inserted in anodized aluminum oxide nanopores; PMPP = Polystyrene microplastic particles; Au@AgNPs = Core(Au)-shell(Ag) nanoparticles; GNFP = Glass nanofibrous paper; MA = Methamphetamine; ACE2 = Angiotensin converting enzyme 2; HAV = Hepatitis A virus; PS@Ag@ZIF-8 = Polystyrene beads@silver@zeolitic imidazolate framework-8; Gr = Graphene; Ph-AgNPs = Phenylacetylene functionalized AgNPs; NPP-NS = Nano-pine-pollen nanostructure; AuNSs-PEG = gold nanostars coated with polyethylene glycol).

SERS Substrates	Sample	Pollutants	LOD	Refs.
Self-assembled AuNPs	Wastewater	Quinoline	5 μg/L	[69]
r-Ag/Au fiber	Household wastewater	Benzidine	5 μg/L	[70]
Fe${}_{3}$O${}_{4}$NR@AuNPs	Water	CIP	$10^{-7}$ M	[71]
Ag–Cu–PLA disks	Lake water	CMIT	10 mg/L	[72]
Au nanoparticles	Refinery process water	MEA	1.8 mg/L	[73]
AuMNPs	Water	Ibuprofen	$10^{-8}$ M	[74]
AgNPs on DE film	Wastewater	Fentanyl	0.8 μg/L	[75]
AuNSs@Ag@AAO	Tap, river and sea water	PMPP	50 mg/L	[76]
Au@AgNPs on GNFP	Wastewater	MA	7.2 ng/L	[77]
ACE2@AgNRs array	Various waters	SARS-CoV-2	–	[78]
Au pyramidal nanoholes	Water	HAV	13 ng/L	[79]
PS@Ag@ZIF-8	Tap water	Cu${}^{2+}$	$10^{-7}$ M	[80]
Gr/Au/Ag/GaN	Water	Pb${}^{2+}$	$4.3\times 10^{-12}$ M	[81]
Ph-AgNPs	Lake water	Hg${}^{2+}$	$8.8\times 10^{-11}$ M	[82]
NPP-NS	Drinking water	Cd${}^{2+}$	$10^{-11}$ M	[83]
AuNSs-PEG	Seawater	Hg${}^{2+}$	0.2 μg/L	[84]

## Data Availability

Not applicable.
